# Synthesis and Characterization of Microwave-Assisted Copolymer Membranes of Poly(vinyl alcohol)-g-starch-methacrylate and Their Evaluation for Gas Transport Properties

**DOI:** 10.3390/polym14020350

**Published:** 2022-01-17

**Authors:** Mallikarjunagouda Patil, Shridhar N. Mathad, Arun Y. Patil, Muhammad Nadeem Arshad, Hajar Saeed Alorfi, Madhu Puttegowda, Abdullah M. Asiri, Anish Khan, Naved Azum

**Affiliations:** 1 Bharat Ratna Prof. CNR Rao Research Centre, Basaveshwar Science College, Bagalkot 587101, Karnataka, India; mallupatil04@gmail.com; 2 Department of Engineering Physics, KLE Institute of Technology, Hubballi-580030, Karnataka, India; physicssiddu@gmail.com; 3 School of Mechanical Engineering, KLE Technological University, Vidya Nagar, Hubballi-580031, Karnataka, India; patilarun7@gmail.com; 4 Center of Excellence for Advanced Material Research, King Abdulaziz University, Jeddah 21589, Saudi Arabia; mnachemist@hotmail.com (M.N.A.); aasiri2@gmail.com (A.M.A.); anishkhan97@gmail.com (A.K.); 5 Chemistry Department, Faculty of Science, King Abdulaziz University, Jeddah 21589, Saudi Arabia; hsalorfi@gmail.com; 6 Department of Mechanical Engineering, Malnad College of Engineering, Visvesvaraya Technological University, Belagavi, Hassan-573202, Karnataka, India; mputtegowda@gmail.com

**Keywords:** copolymer, gas permeation, membrane, permeability, selectivity

## Abstract

Poly(vinyl alcohol) (PVA) is an excellent membrane-forming polymer and can be modified with potato starch and methyl acrylate monomers to obtain copolymers with improved physical and chemical properties. The study presents the synthesis of poly(vinyl alcohol)-g-starch-poly(methyl acrylate) PVA-g-St-g-PMA copolymers using microwave irradiation technique and potassium persulfate initiator. Solution casting and solvent evaporation methods were adopted for the fabrication of polyvinyl alcohol-g-starch-acrylamide composite membranes. The synthesized graft copolymer was characterized by Fourier transform infrared spectroscopy, scanning electron microscopy, and thermal analysis. The modified nanocomposite membranes were showed very promising results with the parameters permeability and selectivity. The nanocomposite membranes exhibited the advantages of easy handling and reuse.

## 1. Introduction

Mainly ten basic greenhouse gases are identified which include vapors of water (H_2_O), carbon dioxide (CO_2_), methane (CH_4_), and nitrous oxide (N_2_O) that are naturally occurring. Because of Industrial activities Perfluorocarbons (CF_4_, C_2_F_6_), hydrofluorocarbons (CHF_3_, CF_3_CH_2_F, and CH_3_CHF_2_), and sulfur hexafluoride (SF_6_), are present in the atmosphere. Water vapor is the most significant, abundant, and leading greenhouse gas, the second one is the CO_2_ is the second-most important one [1,2,3,4]. It is very necessary to capture CO_2_ and lots of technologies have been developed in past decades. The membrane gas separation technique is a widely accepted technique across the globe for the separation of the gases from its mixture. Now a day’s it is very necessary for most industries is that treatment of fumes from coal-fired plants especially aiming for CO_2_ removal to reduce pollution and decrease the greenhouse effect. The focus is also given to other applications such as the separation and purification of economically important gases such as H_2_, O_2,_ and CH_4_ from natural gas. So far the most common methods adopted by the people to separate the gases are (a) Separation with solvent/sorbents (b) Separation by cryogenic distillation and (c) Separation with Membranes. Among all the techniques adopted, the membrane separation technique is the most fusible and economic technique so far considered [5,6,7].

In membrane-based gas separation, the principle adopted is that the gas separation across the membrane on the affinity towards the membrane surface, causing one gas to permeate faster than the other. Membrane gas separation is the fastest-growing field in membrane-based separation technology this is due to the availability of a wide variety of materials to which the membrane could be tailored, including nanocomposites, zeolites, ceramic, and metal-containing materials [6,7,8]. Carbon dioxide separation from natural gas streams and other emission sources, especially flue gas from power plants and petroleum/chemical industries, has become a necessary field of research owing to the rising concern for global warming caused by the effects of greenhouse gases [9]. The polymeric membranes found applications in modern-day separation and purification industries. Polymers play a vital role in membrane-based separation technology. Other than polymers in the membranes ceramics, metals are taking their next role [9].

The development of the efficient membrane is the main challenge in gas separation. The main intention of modifying or improving the membrane is to enhance permeability and selectivity. In membrane-based separation technology, the development of an efficient membrane is the crucial focus of research. The main goal of developing a membrane is to enhance permeability and selectivity [10,11,12]. Several unmodified and modified membranes were employed in the CO_2_ separation from its mixture gases. Since the beginning, cellulose acetate/triacetate and polyimide polymers were exploited as membranes since the high-performance fixed site-carrier (FSC) membranes and are having the ability to show greater enrichment for CO_2_/N_2_ and CO_2_/CH_4_ gases [9,13].

The grafting method, via the microwave radiation technique, is very tremendous in phrases of time consumption, cost-effectiveness, and environmental friendliness. Graft copolymerization of methyl methacrylate (MMA) onto cellulose via chemical and radiation techniques is properly investigated [14]. Microwave irradiation (MWR) offers a speedy energy switch and high-energy efficiency. Microwave radiation (MWR) assists in direct heating of solvents and reactants. Owing to this fascinating heating mechanism, which is genuinely distinct from other traditional heating, selective heating can be performed and many reactions can be accelerated [15]. By using MWR we have several advantages, by using this method it will helps to reduce the level of physicochemical stress occurred during the reaction, in which the monomers or polymers are not reacted at some points in the conventional method. Microwave technique employs electromagnetic radiation, which will penetrate deeply inside the core of the molecule as a result molecule, will get extra energy and they oscillate. Microwave radiations will gets activated polar molecules like water present inside the polymer matrix and these water molecules as solvent will also gains energy and do part during the course of reaction by get heated. Several researchers have been studied the grafting of vinyl monomers on to several natural polymers by using MWR’s in order to improve the pristine polymer property [16].

In order to improve the nascent polymeric membranes permeation property and selectivity it is necessary to go for the modification of the membrane. In view of attempting the better performance of the membrane, several attempts were made by the researchers. In the present study the starch a cellulosic family natural polymer and methyl acrylate monomers were grafted in aqueous solution in presence of PVA and the change in gas transport property was observed.

## 2. Experimental

### 2.1. Materials and Method

IFB 30 L Convection Microwave Oven Model no 30BRC2 (IFB, Bangalore, India); 2200 Watts, Domestic microwave oven having Operation Frequency 2450 MHz microwave frequency and power output from 0 to 900 W with continuous adjustment was used for all the experiments. All the reagents used were of analytical grade. Distilled water was used throughout the study.

Polyvinyl alcohol (mol. wt. 125,000) and potato starch were obtained from S.d. fine chem. Limited, Mumbai; India was used without further purification. K_2_S_2_O_8_ was purchased from Merck, Bangalore, India. Double distilled water was used throughout the experiment.

### 2.2. Synthesis of PVA-g-St-g-PMAcopolymer

5% (*w*/*v*) polyvinyl alcohol solution was prepared by dissolving the desired amount of PVA in 100 mL of distilled water at 70 °C. In a vessel in the water, starch graft PMA solution was prepared by constant stirring with heating at 100 °C to get homogeneous slurry. 0.02 M K_2_S_2_O_8_ was added as an initiator to the prepared slurry with constant stirring at 600 rpm. Once the clear homogeneous mixture was obtained after stirring, the flask was exposed to microwave irradiation in a domestic microwave oven for three minutes at 640 W. After completion of the reaction, the reaction mixture was allowed to cool and the mixture was precipitated with a 3:1 ratio methanol: water mixture. The resulting raw product was filtered using a vacuum pump and washed with water several times and dried in an incubator at 60 °C temperature to a constant weight.

### 2.3. Membrane Fabrication

**Figure 1:**
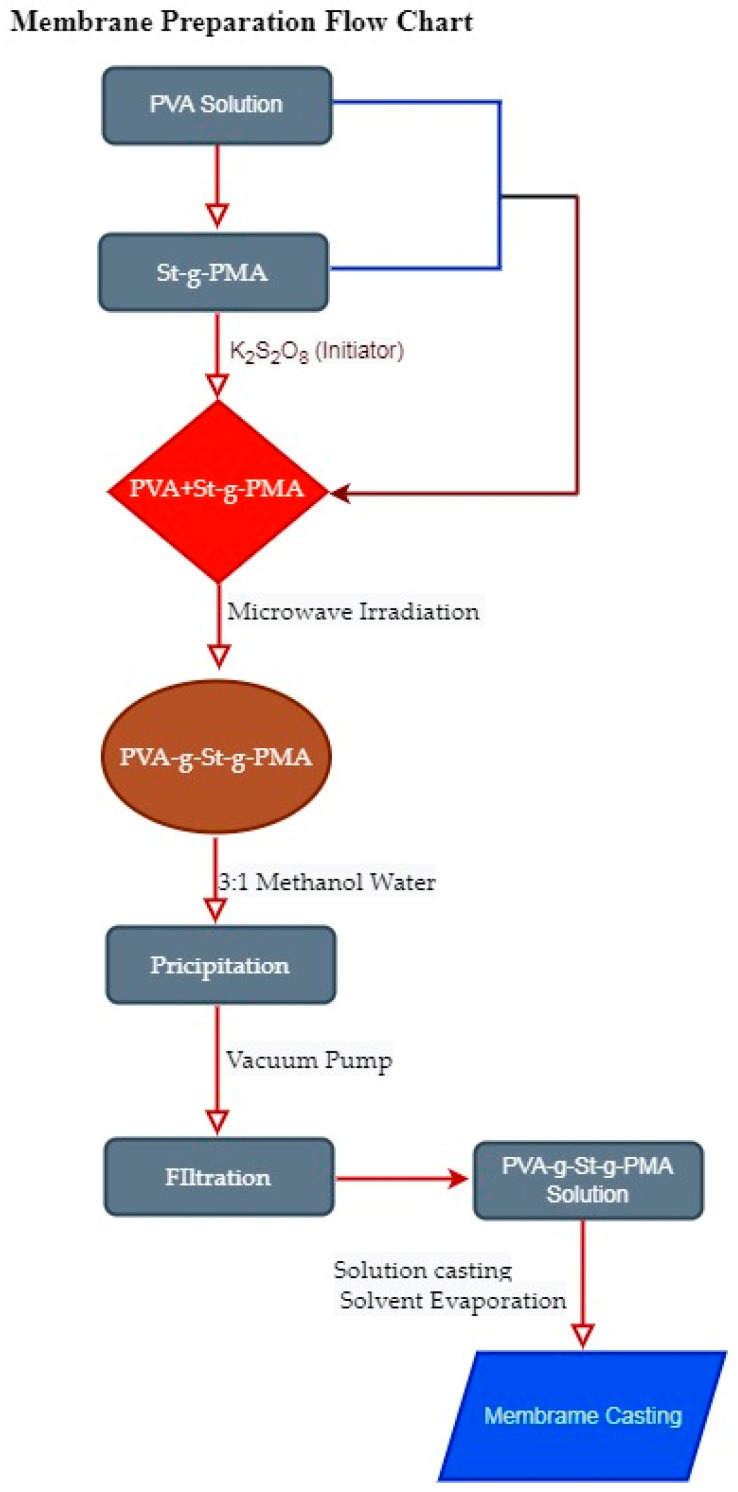
Flow chart of synthesis of PVA-g-St-g-PMA and membrane fabrication.

5 gm of obtained PVA-g-St-g-PMA was dissolved in distilled water under constant stirring. The mixture was stirred for 12 h to completely homogenize. The copolymer was homogeneous; the solution will become highly viscous. The resultant mixture was cast into a clean and dry glass plate. Once the casted solution was dried, the membrane was peeled off from the glass plate and stored in a dry place. PVA-g-St-g-PMA. The detailed step from synthesis of PVA-g-St-g-PMA and membrane fabrication is shown in the Figure 1.

### 2.4. Permeability Measurements

Single gas permeabilities for N_2_, CO_2_, were evaluated using a gas permeameter. The Permeameter consists of a stainless steel permeation cell that separates upstream (feed side) and downstream (permeate side). A pressure transducer is connected to the downstream side to measure the pressure change with time on the permeate side. This cell exposes a membrane area of 13.302 cm^2^ to the gas.

The constant-volume variable pressure method was used to measure the permeation of the pure gas. The pressure increase with time was plotted from the raw data. The gas permeability is calculated based on the following equation:

(1) $$P=\frac{Vl}{A}\frac{T_{0}}{P_{f}P_{0}T}\left[{\left(\frac{dp}{dt}\right)}_{ss}-{\left(\frac{dp}{dt}\right)}_{leak}\right]$$

where:

- *P* is the permeability of the gas through the membrane (barrer), (1 Barrer = 10^−10^ cm^3^ (STP) cm cm^−2^ s^−1^ cmHg^−1^).
- *V* is the permeate volume (cm^3^).
- *l* is the thickness of the membrane layer (cm).
- *A* is the effective area of the membrane (cm^2^).
- *P_f_* is the feed pressure (cmHg).
- *P*_0_ is the pressure at standard state (76 cmHg).
- *T* is the absolute operating temperature (K).
- *T*_0_ is the temperature at standard state (273.15 K).
- (*dp*/*dt*)*_ss_* is the rate of pressure increase in the permeate side at the steady state (cmHg s^−1^) under the feed pressure.
- (*dp*/*dt*)*_leak_* is the pressure increase in the permeate side under vacuum (leakage pressure increase).

The ideal selectivity α*_A_*_/*B*_ of gas pairs, *A* and *B*, was calculated. It is defined as the ratio of their permeably:

(2) $$\alpha_{A/B}=\frac{P_{A}}{P_{B}}$$

## 3. Characterization

### 3.1. FTIR

**Figure 2:**
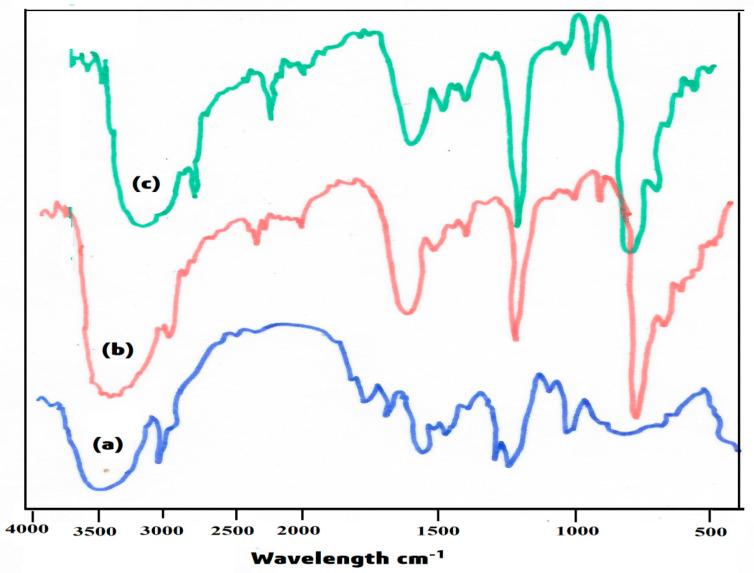
FTIR Spectra of (**a**) Pure PVA (**b**) St-g-PMA and (**c**) PVA-g- St-g-PMA.

FTIR (Fourier transform infrared spectroscopy) spectra of plain PVA and PVA-g-St-g-PMA were taken in the range between 4000 and 400 cm^−1^ using Schemadzu (Chiyoda-ku, Tokyo, Japan), Model: IR-Affinity-I FTIR spectrometer. Copolymeric membrane samples were grounded well with spectroscopic grade KBr and pellets were formed by pressing under the hydraulic pressure of 400–450 kg/cm^2^. FTIR spectral curves are displayed. The FTIR curves are displayed in Figure 2.

### 3.2. Thermogravimetric Analysis

**Figure 3:**
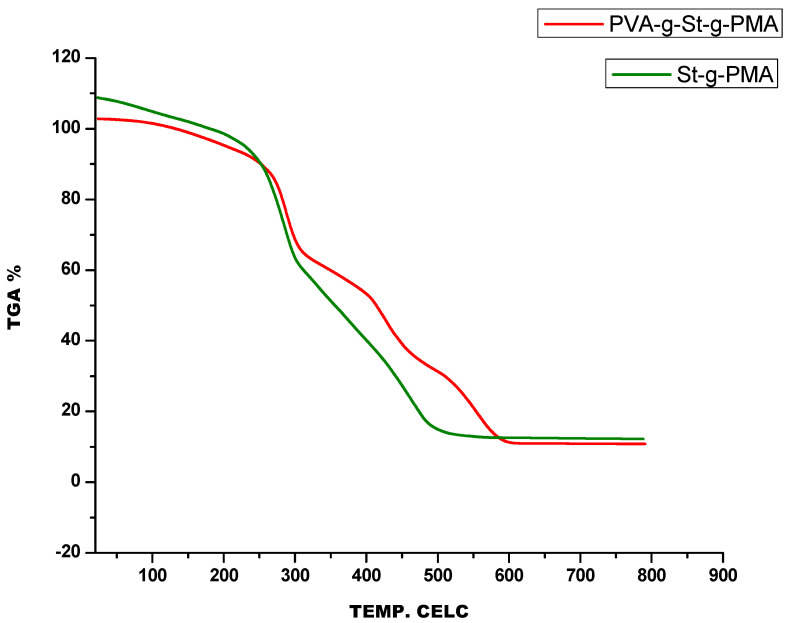
Comparative TGA graph of St-g-PMA and PVA-g-St-g-PMA.

The thermal analysis of the plain PVA and prepared copolymer were scanned by Thermogravimetric Analysis (TGA) Mettler Toledo model TGA 2 (SF). The melting studies were performed in the temperature range of 25 °C to 800 °C at a heating rate of 10 °C/min in an oxygen environment. The TGA curves are displayed in Figure 3.

### 3.3. Scanning Electron Microscopy (SEM) Studies

Surface morphology of plain PVA and PVA-g-St-g-PMA matrix and prepared gel matrix was evaluated with a model Jeol, Model 7900F Scanning Electron Microscope SEM (Akishima, Tokyo, Japan) after sputter coating of gold on the specimen surface.

### 3.4. Mechanical Properties

Tensile strength and ultimate elongation were measured for all the prepared membranes. Tensile tests were performed with a universal testing machine (UTM) (Lloyd, Worcester, UK) according to the test method ASTM D 412 by using a 5.0 N load cell with a crosshead speed of 100 mm/min. An average of 5 tests for each polymer membrane sample was considered.

## 4. Result and Discussion

### 4.1. FTIR Spectroscopy

The FTIR spectra of pure PVA curve in Figure 2a showed a broad peak around 3426.1 cm^−^^1^ demonstrating the occurrence of –OH group with intermolecular hydrogen bond in single bridge compounds and the peaks appeared at 3022.1 and 1217.1 cm^−^^1^ are shown C-H stretching and C-H bending representing the presence of hydrocarbon chromophore in PVA matrix.

The FTIR spectrum of synthesized membrane i.e., PVA-g-St-g-PMA matrix is shown in Figure 2c, which showed a peak at 3434.3 cm^−^^1^, representing the presence of intermolecular hydroxyl groups. Apart from this peak, the polymer matrix is showing peaks at 3025.1 cm^−^^1^ and 1217.8 cm^−^^1^ demonstrating the presence of hydrocarbon chromophore. The absence of characteristic peak of the carboxylic group at 1746.1 cm^−^^1^ indicated the absence of >C=O groups from this. It can be concluded that the carboxylic groups of St-g-PMA Figure 2b have been utilized for grafting and facilitated that PVA has been successfully grafted onto the St-g-PMA backbone.

### 4.2. Thermogravimetric Analysis

The thermal properties like thermal stability and degradation of St-g-PMA and PVA-g-St-g-PMA samples were examined by TGA under atmospheric conditions. The results of the St-g-PMA and PVA-g-St-g-PMA matrix are shown in the TGA curve and are plotted in Figure 3. The Thermogram of PVA-g-St-g-PMA shows three stages of weight loss. Initially, up to 100 °C, 4% of weight loss has been observed and that may be attributed to the loss of Physico-chemical bound moisture. The second decomposition has been taken place at temperatures 200 °C, 300 °C, and 500 °C with corresponding weight losses of about 9.4, 41.8, and 86.2% respectively. In the literature, it was reported that St-g-PMA decomposition occurred in two stages and it was thermally stable up to 265 °C with a weight loss of around 15% [17,18]. The decomposition products of pure PVA were also reported previously [19,20].

### 4.3. Scanning Electron Microscopy(SEM) Study

**Figure 4:**
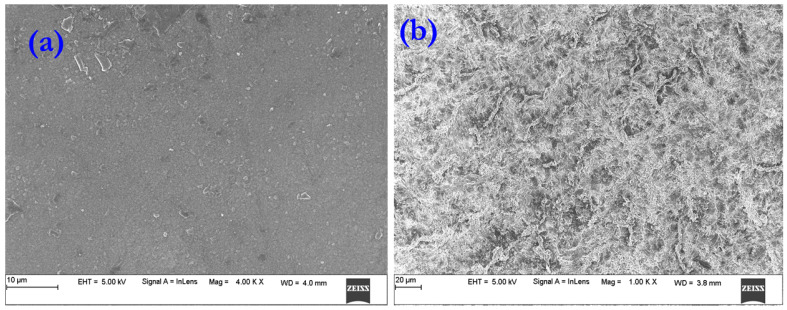
SEM images of (**a**) Plain PVA membrane and (**b**) PVA-g-St-g-PMA membrane.

SEM image of the surface of the plain PVA membrane and PVA-g-St-g-PMA membrane is shown in Figure 4a. Notice that the polyvinyl alcohol polymer matrix surface appears smooth. In Figure 4b represents PVA-g-St-g-PMA, containing voids as shown in Figure when compared to the agglomerated surface of St-g-PMA (Figure 4b).

### 4.4. Mechanical Properties

Mechanical properties were recorded for all the prepared membranes as per ASTM D 412 and are tabulated in Table 1. The highest tensile strength is observed for the plain PVA membrane, while on the other hand ultimate elongation is least for plain PVA and showed maximum value for the copolymer i.e., 863.6. This data shows that the increased flexibility of the membrane materials in the grafted polymeric membrane.

The prepared graft polymeric films are elastic in nature and the reason behind is that the formation of internal hydrogen bonding and increase in the intermolecular distance [21]. The obtained results were confirmed that as the increase of St-g-PMA content in the graft polymeric membrane as the elongation at break increased consequently, this may be the reason that the grafting of St-g-PMA will trap in between the PVA polymeric matrix and acts as the plasticizer. This is the reason for the polymer film flexibility with compromising the tensile strength. One can conclude that this is the reason for a huge gain in the % elongation of PVA-g-St-g-PMA film.

### 4.5. Gas Permeability Studies

**Figure 5:**
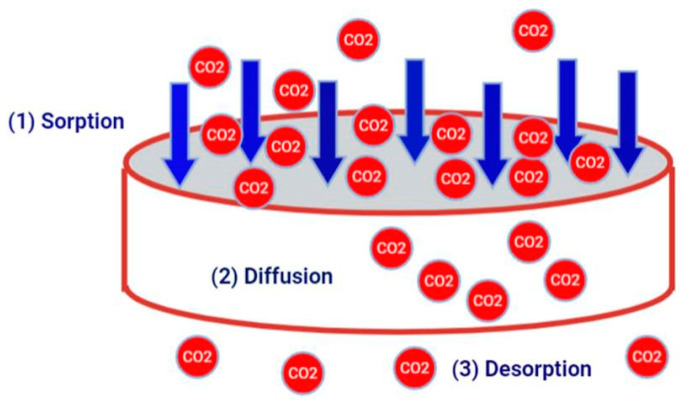
Solution-diffusion model for gas separation.

Gas permeation through the synthesized copolymer is predicted to observe the solution-diffusion mechanism as in any dense polymeric membrane. This mechanism directs the gas permeation behavior through dense polymeric membranes; hence, separation is not only dependent on the kinetic diameter of the molecule but also relies on the chemical interaction between the membrane surface and gas molecules during the diffusion stage. The Solution-diffusion model was illustrated in Figure 5.

**Figure 6:**
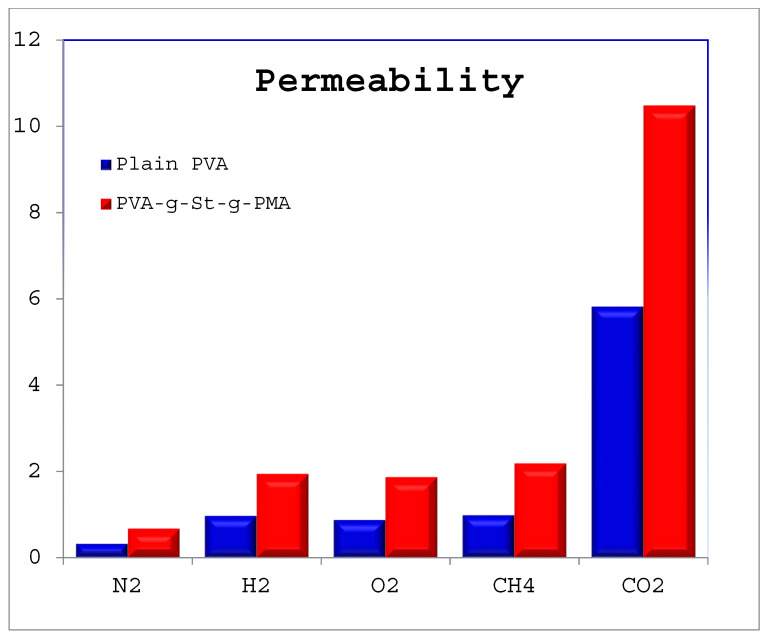
Single gas permeability for CO_2_, H_2_, CH_4_, O_2,_ and N_2_ gases for plain PVA and PVA-g-St-g-PMA membrane.

The prepared membranes were assessed for the gas permeability measurement. Pure gas permeability was measured for N_2_, CH_4_, and CO_2_ gases with the prepared PVA-g-St-g-PMA membranes and are compared with its plain membrane. The permeability values for the tested gases are plotted in Figure 6.

The permeability is mainly due to the affinity of the gaseous molecules towards the top side of the membrane. The permeability of plain PVA is 0.969, 5.816, 0.874, 0.323, and 0.984 for the pure gases H_2_, O_2_, N_2_, CH_4_ respectively. As per the Figure 6, the permeability for PVA-g-St-g-PMA is calculated as 1.941, 10.474, 1.866, 0.674, and 2.186 respectively. From the data plotted in Figure 6, it is observed that plain PVA and grafted polymer membrane show the highest permeability for CO_2_ and least for N_2_ gas. It is also observed that the modified graft copolymer showed good performance for all the measured gases against its plain membrane i.e., plain PVA membrane. Amongst all tested gases the maximum permeability value obtained is for CO_2_ the reason behind this is due to the tetravalent and affinity of the CO_2_ towards the membrane and on the other hand the permeability value for N_2_ is minimum this is because of its low affinity and inertness towards the membrane. The permeability data were plotted as a bar graph in Figure 6.

Permeability result of CO_2_ was greater than the permeability result of N_2_ for all tested membranes. The reason could be interpreted by inferring to the kinetic diameter of the gases. In case of CO_2_, which is acidic by nature, and has 3.3 Å kinetic diameter, and the value is smaller as compared with the kinetic diameter of the N_2_ having 3.64 Å [22]. Gases with smaller kinetic diameter and highly condensable, for instance CO_2_, will ultimately diffuse faster than gases with bulky kinetic diameter [23]. Apart from the kinetic sizes of the gases, the permeability of gases greatly influenced by the interaction of gaseous molecules with polymer matrix. CO_2_ gas has high interaction with membrane matrix and the reason is the presence of hydroxyl functional group (–OH) on the membrane matrix. The interaction will lead to increased solubility of CO_2_ in the membrane surface, while enhances the diffusivity of CO_2_ and, thus, results in increased permeability compared to N_2_ gas [24,25].

### 4.6. Selectivity

**Figure 7:**
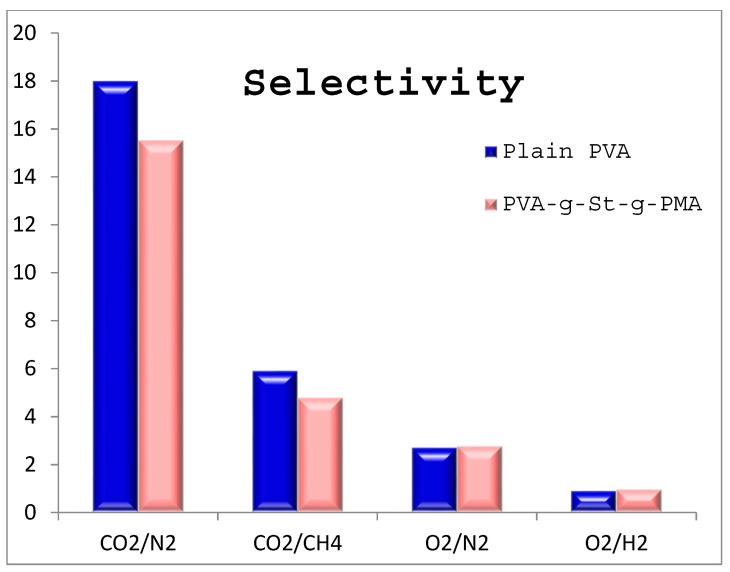
Selectivity data for CO_2_/N_2_, CO_2_/N_2_, O_2_/N_2_, O_2_/H_2_ gases for plain PVA and PVA-g-St-g-PMA membrane.

A comparative selectivity has been calculated and is plotted as a chart in Figure 7. The case of the selectivity of CO_2_/N_2_ has shown the highest selectivity and lowest for O_2_/H_2_. The obtained results suggest that the prepared membranes were suitable for the CO_2_/N_2_ separation. In all the cases synthesized PVA-g-St-g-PMA copolymer was performed well in terms of selectivity.

Figure 7 gives the idea about the PVA-g-St-g-PMA membrane performance in terms of selectivity. Amongst the calculated selectivity, highest value of selectivity is obtained for CO_2_/N_2_ gas i.e., 15.6. Other combination of gases i.e., CO_2_/CH_4_, O_2_/N_2_ and O_2_/H_2_ the selectivity value decreased 4.8, 2.8 and 0.96 respectively. The reason for the decrease in the selectivity value is due to reduction in partial pressure gradient and due to coupling effect [26,27]. This effect is explained clearly in two different ways [28,29], one is the plasticization effect of the membrane. Secondly the PVA-g-St-g-PMA adsorbs the gaseous molecules and fails to desorb the same.

## 5. Conclusions

PVA-g-St-g-PMA matrix was synthesized under microwave irradiation. A very high yield was obtained. The present study demonstrates improvement in gas separation properties of the graft copolymeric membranes compared to the plain PVA membrane. The membranes were much affinity towards CO_2_ and showed the least performance towards N_2_. The prepared copolymer is a better candidate for the CO_2_/N_2_ and CO_2_/CH_4_ gas separation and furthermore, studies require to explore the further membrane performances.

## Figures and Tables

**Table 1:** Mechanical property of all polymeric membranes.

Membrane	Tensile Strength M.Pa ± S.D	% Elongation ± S.D
Plain PVA Membrane	84.7 ± 0.5	91.4 ± 0.3
PVA-g-St-g-PMA	71.0 ± 0.4	863.6 ± 0.5

## Data Availability

Not Applicable.
